# Considerations and best practices in animal science 16S ribosomal RNA gene sequencing microbiome studies

**DOI:** 10.1093/jas/skab346

**Published:** 2022-02-01

**Authors:** Margaret D Weinroth, Aeriel D Belk, Chris Dean, Noelle Noyes, Dana K Dittoe, Michael J Rothrock, Steven C Ricke, Phillip R Myer, Madison T Henniger, Gustavo A Ramírez, Brian B Oakley, Katie Lynn Summers, Asha M Miles, Taylor B Ault-Seay, Zhongtang Yu, Jessica L Metcalf, James E Wells

**Affiliations:** 1 U.S. Department of Agriculture, Agricultural Research Service, U.S. National Poultry Research Center (USNPRC), Athens, GA 30605, USA; 2 Department of Animal Sciences, Colorado State University, Fort Collins, CO 80524, USA; 3 Joint Institute of Food Safety and Applied Nutrition, University of Maryland, College Park, MD 20740, USA; 4 Department of Veterinary Population Medicine, University of Minnesota, St. Paul, MN 55108, USA; 5 Meat Science and Animal Biologics Discovery Program, Department of Animal and Dairy Sciences, University of Wisconsin, Madison, WI 53706, USA; 6 Department of Animal Science, University of Tennessee, Knoxville, TN 37996, USA; 7 College of Veterinary Medicine, Western University of Health Sciences, Pomona, CA 91766, USA; 8 U.S. Department of Agriculture, Agricultural Research Service, Beltsville Agricultural Research Center (BARC), Beltsville, MD 20705, USA; 9 Department of Animal Sciences, The Ohio State University, Columbus, OH 43210, USA; 10 USDA ARS US Meat Animal Research Center (USMARC), Clay Center, NE 68933, USA

**Keywords:** 16S rRNA gene, amplicon sequencing, bacteriome, bioinformatics, microbiome

## Abstract

Microbiome studies in animal science using 16S rRNA gene sequencing have become increasingly common in recent years as sequencing costs continue to fall and bioinformatic tools become more powerful and user-friendly. The combination of molecular biology, microbiology, microbial ecology, computer science, and bioinformatics—in addition to the traditional considerations when conducting an animal science study—makes microbiome studies sometimes intimidating due to the intersection of different fields. The objective of this review is to serve as a jumping-off point for those animal scientists less familiar with 16S rRNA gene sequencing and analyses and to bring up common issues and concerns that arise when planning an animal microbiome study from design through analysis. This review includes an overview of 16S rRNA gene sequencing, its advantages, and its limitations; experimental design considerations such as study design, sample size, sample pooling, and sample locations; wet lab considerations such as field handing, microbial cell lysis, low biomass samples, library preparation, and sequencing controls; and computational considerations such as identification of contamination, accounting for uneven sequencing depth, constructing diversity metrics, assigning taxonomy, differential abundance testing, and, finally, data availability. In addition to general considerations, we highlight some special considerations by species and sample type.

## Introduction

**Figure 1.**
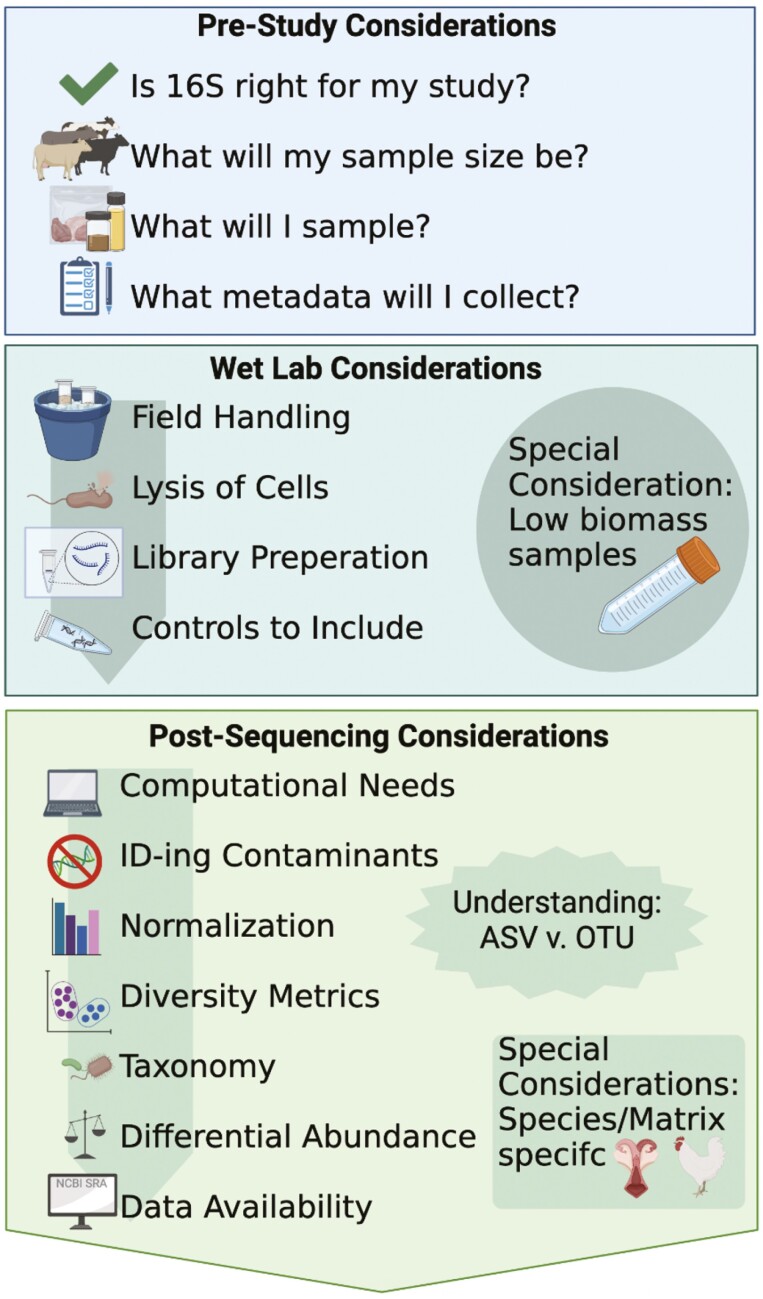
Overview of considerations when conducting a 16S rRNA gene sequencing study. Created with Biorender.com.

Nearly all environments on Earth are inhabited by complex communities of microorganisms. While these environments have traditionally been studied by obtaining and classifying pure microbiological laboratory cultures, culture-based studies limit scientific discovery to the microbes that can be grown in laboratory conditions, thus constraining our ability to fully characterize these microbial communities. The discrepancy between the viable colonies on an agar plate and the count of bacteria under a microscope has been termed the “great plate count anomaly” (Staley and Konopka, 1985) with some estimates approximating that only 1% of bacteria can be cultured with standard techniques (Hofer, 2018). The development of culture-independent technologies, specifically next-generation sequencing (**NGS**) coupled with bioinformatic analyses, has enabled the comprehensive characterization of complex microbial communities or microbiomes. One example of the power of NGS paired with bioinformatic advancements is when Nielsen et al. (2014) examined 396 human gut microbiome samples and identified that 181 new microbial genomes corresponded to previously undescribed species. The parallel evolution of various “-omics” technologies has allowed us to answer key questions about microbiomes, including which microbes and genes are present, what they are capable of doing, and what functions they are performing (Addis et al., 2016). The least financially and computationally expensive of these approaches is 16S ribosomal RNA gene sequencing (sometimes shortened to 16S rRNA or just 16S). This approach targets specific genes that allow taxonomic classification and diversity estimation of the bacterial and archaeal microbiome (this approach does not include *all* microorganisms in a sample; e.g., it cannot capture fungal, viral, protozoal, or eukaryotic organisms). Over the past 10 yr, the combination of lowered DNA sequencing costs and increased accessibility to bioinformatic tools has allowed more animal scientists to incorporate 16S rRNA gene sequencing into their research programs. In fact, while in 2010, the *Journal of Animal Science* only had four research articles that used the word “microbiome,” and by 2020, that number had climbed to 184 publications (72 full-length articles and 112 abstracts) in just 1 yr. As more researchers incorporate 16S rRNA gene sequencing into their programs, they should be familiar with microbiome-specific vocabulary (Table 1) and experimental considerations (Figure 1) to produce high-quality, reproducible results. The purpose of this review is to provide a brief tutorial on the use of 16S rRNA gene sequencing methods and analysis and also to remove barriers to performing microbiome studies in animal sciences.

**Table 1.** Glossary of commonly used microbiome terms

Term	Definition
16S rRNA gene	Gene encoding the RNA component of the 30S subunit of a prokaryotic ribosome; ubiquitous to bacteria and archaea
Alpha diversity	The variance within a sample, used to evaluate the number of different species (usually represented by the number of ASVs) in each sample
Amplicon	The fragment of DNA resulting from a primer set after amplification using PCR
ASV	Amplicon Sequence Variant: individual sequence variants differing by as little as one nucleotide with no fixed dissimilarity threshold
Barcoding	Unique DNA sequences attached to broad range primers before amplification. These unique barcodes allow different samples to be pooled and sequenced together in the same run and later separated during analysis (see demultiplexing)
Beta diversity	The variance between samples, usually expressed as a distance matrix
Demultiplexing	Separation of sequencing reads from a sequenced pooled library by unique barcodes and assignment to the corresponding samples
Evenness	Balance of the features (ASVs, species, etc.) within a sample
Extraction Controls	Blank or non-DNA samples (such as an empty sponge) added to a study to assess background laboratory contamination (see also library controls and NTC)
Feature Table	Also known as a count table (as when using OTUs, OTU Tables). Table that contains the number of sequences counted for each feature (ASV or OTU most commonly), per sample in a matrix
GUI	Graphical User Interface: Computer program that allows users to “point-and-click” as opposed to the command line
HPC	High-performance computing cluster: More powerful computer than a local system many universities have shared HPC for high computational jobs
Library Controls	Controls included with PCR libraries to assess primer performance and contamination (see NTC)
Library pooling	Combines barcoded DNA during library preparation to make one pooled sample of DNA for sequencing. Individual identity is maintained through barcoding
Long-read	DNA fragments generated that range in length from 5 kb+, most commonly on a PacBio or Nanopore sequencer
Metadata	Data that represent biological data collected, describing the information surrounding the data to provide context for analysis and interpretation
Metagenome	Refers to all the genomes represented in a biological mixture
Mock Community	A bacterial mixture (internally generated or commercially available) with known proportions of bacterial to assess sequencing quality and act as a positive control
NTC	No-template controls: Controls included with PCR libraries to assess primer performance and contamination (see Library control)
Normalization	Transformation of raw read numbers to account for uneven read numbers— usually in this method, the ASV numbers are multiplied by a value or proportion.
OTU	Operational Taxonomic Unit: clusters of sequencing reads that differ by less than a fixed dissimilarity threshold (usually 3%) see also ASV
Paired-end sequencing	A DNA fragment is sequenced from both ends (usually 100- to 300-bp long)
Phylogenetic trees	Tree representative of the evolutionary relationship between sequences in the sample can be constructed de novo from only sequences in a dataset or compared with a reference tree
Pipeline	A collection of tools, programs, and other codes that are run in succession to produce results (common pipelines include QIIME2, Mothur, and RCP)
Rarifying	Randomly subsampling ASVs or OTUs within a sample without replacement to a preselected depth
Raw reads	Number of reads generated from each sample; due to sequencing inefficiency, this number will not be the same across samples and thus normalization is needed
Relative abundance	Percentage of a total population attributed to one taxon such as phyla or species in relation to other features in the community
Richness	Number of different species within a sample, regardless of how they are distributed
Sample pooling	Combination of raw sample material (such as equal amounts of rumen fluid) or DNA (not to be confused with library pooling, here no individual identity is maintained)
Short read	DNA fragments generated that range in length from 75 to 300 bp, most commonly on an Illumina sequencer
Shotgun metagenomics	All DNA within a mixed microbe environment, fragmented, and sequenced. Differs from the amplicon 16S approach as it is not amplifying one target but any piece of the genome.
Single-end sequencing	A fragment is sequenced only from one end to the other (usually ~75- to 100-bp long)
Taxonomy	Represents the identification and classification of each microorganism, represented by an ASV, present in the community; this is distinct from phylogeny, which represents evolutionary relatedness of the ASVs
V1 to V9	Hypervariable regions studied on the 16S rRNA gene
V4	A common hypervariable region for 16S studies, also the target for the Earth Microbiome Project

## Overview of the Method

### What is 16S rRNA gene sequencing?

**Figure 2.**
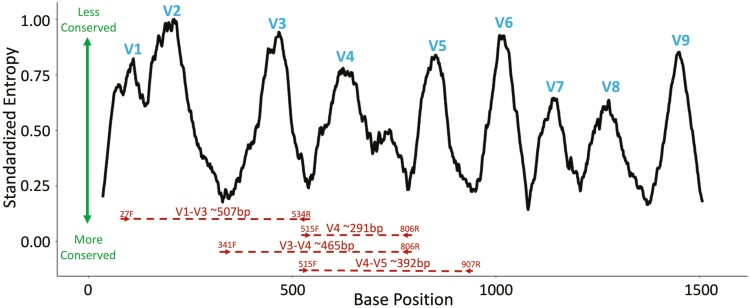
Illustration of conserved and variable regions of the 16S rRNA gene. Hypervariable regions are labeled in blue with conserved regions indicated by low entropy. In red, four commonly used primer pairs are highlighted. The figure was made by using Shannon entropy data generated from Johnson et al. (2019) (https://github.com/TheJacksonLaboratory/weinstock_full_length_16s) and ggplot2 in R (v. 4.0.2).

Using the ribosomal RNA gene as a marker for determining evolutionary relatedness was first proposed by Carl Woese due to its ubiquity in all organisms (Olsen and Woese, 1993). The 16S rRNA gene sequencing method involves targeting only a small fraction of microbial DNA, which provides useful insights into the diversity and identification of microbial communities. The 16S rRNA gene codes for the RNA component of the 30S subunit of a prokaryotic ribosome (bacterial ribosomes are composed of a large [50S] and a small [30S] subunits). This gene, which is ubiquitous in bacteria and archaea, has been described as a “molecular clock” because it allows for phylogeny determination and species divergence due to its structure and activity in cellular function (Duchêne et al., 2016). The 16S rRNA gene is around ~1,550 base pairs (bp) long and is composed of eight highly conserved regions and nine hypervariable regions (with these regions named V1 to V9; Figure 2) (Clarridge, 2004). When conducting a 16S rRNA gene sequencing study, one or several hypervariable regions are amplified using broad-range primers that each bind to a conserved region and are sequenced. Then, the information in these regions is used to reconstruct the taxonomic composition (done by comparing the sequences to databases of known organisms) and diversity present within the sample. Depending on the application, appropriate phylogenetic classifications can sometimes be made from 16S rRNA hypervariable fragments as small as 100 bp, making popular and affordable short-read sequencing platforms (e.g., Illumina) suitable for microbiome analysis (Caporaso et al., 2011). While it is possible to sequence the entire length of the 16S rRNA gene for more information, this requires greater investments of time and money, which can undermine the advantages of this approach to high-throughput microbiome sequencing. Likewise, sequencing across rRNA genes to include intergenic regions has proven useful for strain typing bacterial species (Johnson et al., 2019) but requires even greater investments of time and money. In summary, modern microbiome analysis using 16S rRNA gene sequencing is affordable (often significantly less than US $50 a sample) and can provide a culture-independent survey of the bacterial and archaeal community within a sample. 

### Uses and limitations of 16S rRNA gene sequencing

Before pursuing a particular approach for microbiome analysis, it is important to consider your research questions and whether 16S rRNA gene sequencing will be able to answer them. For example,16S rRNA gene sequencing cannot describe metabolic potential or activity of a microbial community. This is because 16S rRNA gene is a housekeeping gene found in all prokaryotes and any sequence variability only indicates phylogenetic divergence, which may or may not correlate with metabolic potential, virulence, antibiotic resistance, etc. Additionally, the 16S rRNA region sequenced may not contain sufficient sequence variability to distinguish species or strains (e.g., a pathogenic vs. a commensal strain of *Escherichia coli*).

A further limitation of sequencing-based approaches is the arbitrary “total” imposed by the sequencer, such that the total absolute microbial load, or abundance, is not reflected. A measurement of total bacterial load would require quantitative methods, such as quantitative polymerase chain reaction (qPCR), and cannot be obtained by 16S rRNA gene sequencing alone (Gloor et al., 2017; Ganda et al., 2021). Data generated by 16S rRNA gene sequencing can be thought of as a survey or “taking attendance” as it provides information on which microbes are present and in what relative abundance. This also allows the use of diversity estimates to describe the microbial ecology of a single sample and to compare similarities and differences among many samples. One known shortcoming to this approach is that different organisms have different copy numbers of the 16S rRNA gene in their genome, so relative abundance is biased toward those with higher copy numbers of the 16S rRNA genes in their genome; but while this is a known limitation, tools to correct this issue have not produced reproducible consistent results and the common practice is to not adjust for 16S rRNA gene copy number by bacterium (Louca et al., 2018). Also of note, if you are looking for a specific bacterium, it would be more advantageous to use specific primers as opposed to relying on the broad range primers, as low levels of the bacterium of interest could be missed with 16S rRNA gene sequencing alone, resulting in a false negative.

Another important caveat to DNA-based 16S rRNA gene sequencing is that it cannot distinguish whether microbes in a sample are dead or alive, which can be a relevant issue in agricultural sciences. For example, applying 16S rRNA gene sequencing to soil sterilized by steaming may suggest highly diverse microbial communities in these matrices, when in fact most of those microbes are dead. Researchers interested in microbial activity might prefer RNA-based or metabolomic approaches to answer those questions or the use of a different set of tools.

## General Experimental Considerations

### Sample size, statistical power, and experimental unit

Although microbiome studies routinely analyze many dependent variables simultaneously (e.g., all the bacterial taxa present in a sample) as opposed to just one (such as rate of gain and carcass size), many of the same experimental design considerations must be evaluated, such as effect and sample size, experimental unit, and study design. While some papers report methods for power analysis for microbiome studies (Kelly et al., 2015; Chen, 2020), these tools have limitations such as the metrics of measurement or are only able to be implemented for studies with a specific design (such as case–control). Unfortunately, due to the complexity of microbiome studies, such as sequencing depth, bioinformatic tools, rare taxa, and unknown effect size, power analysis remains rare in microbiome studies (Debelius et al., 2016). If previous studies have been conducted similar to the study you wish to conduct (unfortunately still uncommon for most animal sciences questions), or if you have the ability to generate a pilot sample set, a better idea of sample and effect size could be obtained. It is likely that as more microbiome data are acquired and publicly shared, sample size and power may become more estimable (McDonald et al., 2015). The experimental and sampling unit (i.e., one animal and one fecal grab) should also be determined prior to the start of the study, with special consideration to the pooling of samples (see below). Finally, although a single sample may produce tens of thousands of sequencing reads, it is still only one sample and best practices in experimental design, and biological replication must still be followed. An investigator must still be aware of limits of sample size and degrees of freedom and not subject microbiome data to overly complex study design—this is especially important given that many microbiome studies have smaller sample sizes due to cost restraints.

### Sample pooling

A specific consideration that is directly tied to sample size is pooling of samples. Samples can be pooled by raw material (such as equal volumes of rumen fluid, feces, or meat) or after DNA extraction such as in equal weights or volumes. Pooling not only can have advantages such as experimental units being more representative of a microbiome (such as 20 fecal grabs from a pen vs. one) and cost but also come with the concern that pooling could mask differences among individual animals or groups (Hamady and Knight, 2009). When pooling samples, several considerations on animal homology must be contemplated, such as production system, background, region, and husbandry. Production system vertical integration should be considered if appropriate—for example, cattle in a feedlot are likely not from all the same stocker sources, whereas chickens in different houses at the same location could be from the same breeder. In fact, in a feedlot study designed to evaluate a feed additive (Huebner et al., 2019), cattle source explained the most of the observed microbiome variations. Knowing the geographical history, diet, and veterinary treatment of animals can help to guide pooling strategy, but oftentimes these host-level factors are unknown. Ideally, pooling would be done across the most homologous group of animals possible such as the same pen, barn, treatment group, or pasture (though pooling across these variables should be accounted for in an experimental design, such as a block). In a best-case scenario, just like any animal science study, applying the treatment to the most homologous group possible will reduce unpartitioned variance. If there is more variation within the pool than between, type II error could be encountered. A tradeoff between smaller experimental units that are more homologous in type vs. larger experimental units that could be more reflective of an entire population needs to be considered when pooling.

Additionally, when pooling, you lose the ability to determine what microbes may be interacting in a single community. For example, if you are interested in which organisms may be associated with the presence of pathogens in a fecal microbiome, pooling across feedlot pen would limit your ability to address that research question if there is a difference between pens. Additionally, pooling may result in rare taxa not being detected as well as possible alterations in alpha and beta diversity. Therefore, like all suggestions made in this review, your choices in designing the sampling scheme should be carefully weighed against your research questions.

### Appropriate sampling location

There are numerous considerations for identifying the correct sampling locations and such considerations should be focused on satisfying the hypothesis or objective of a research project. When choosing a sampling location, the question of biological relevance must be considered; for example, is it appropriate to infer differences in the chicken gastrointestinal tract (**GIT**) microbiota from cloacal swabs, fecal collections, or poultry litter? For example, while there is an association between cloacal swabs and cecal content in broilers, rare taxa in the ceca are not well represented in cloacal swabs (Andreani et al., 2020). Additionally, measuring individual animal phenotypes can be expensive and often impractical in some commercial production settings. Two examples from two different species on choosing a sampling location are 1) deciding which segment of a bovine GIT to sample and 2) determining which sample is the most appropriate representation of the poultry GIT in a broiler study.

In the case of the bovine GIT, where many studies have focused on the reticulorumen due to its primary role in nutrition and metabolism, it is common to use fecal and/or ruminal sampling. Yet, while colorectal or fecal samples are often considered as a proxy for rumen microbial activity, this may be of concern depending on the objective of the study. Using fecal samples maybe warranted if examining factors such as nutrient degradability (Kaltenegger et al., 2021), the impact of a nutritional supplement or diet on fecal pathogen shedding (Jacob et al., 2009), or as an indicator of ruminal acidotic status (Plaizier et al., 2017; Neubauer et al., 2020). However, the rumen microbiome is quite distinct from the lower GIT, and fecal sampling should not typically be considered as a proxy for the ruminal microbiome in many cases. Indeed, researchers have demonstrated that the bacterial communities in the rumen are distinct from those of the small and large intestine, varying significantly in function, phylogenetic diversity, richess, and evenness (Myer et al., 2017; Huang et al., 2020). The absorption of amino acids and fatty acids, digestive processes, post-ruminal degradation of cellulose and starch, and the maturation of the mucosal immune system, all highlight the differences among the segments of the lower GIT in cattle.

This issue is not species specific, and many studies have taken different approaches to understand the microbiome of the chicken GIT. In the past, due to the price associated with euthanizing poultry to obtain digesta samples within the GIT, researchers did not use the GIT but other means such as fecal samples to investigate the microbiota. This effort led to incorrect assumptions of treatment effects on the microbiota (Stanley et al., 2015; Locatelli et al., 2017; Schreuder et al., 2020b; Schreuder et al., 2020a). For example, Schreuder et al. (2020b) determined that it is not practical to use cloacal swabs to infer the environmental impact on the poultry GIT; instead, focus should be on investigating the environmental impact on the GIT microbiota of commercial layers directly over time. Additionally, fecal matter is commonly subjected to sequencing analysis in place of poultry cecal contents as it can be collected over time from the same bird and does not require the bird to be euthanized in order for collection. However, the microbiota of feces is not entirely representative of the cecal microbiota. Stanley et al. (2015) determined that the fecal microbiota of 163 birds across three trials had similar bacteria present within the feces and ceca but not in the same relative abundances.

Therefore, when aiming to determine proxies for ease of sampling, sampling from the feces or other easily accessible matrices may not be appropriate for representing the location of interest. Choosing the correct sample location for these microbiome studies is an essential, and commonly complicated, step in project and experimental design. When determining the sample location for amplicon-based rRNA research, the location must ultimately be based upon the hypothesis and/or objective(s) of the study, as well as knowledge regarding the physiology of the host species and/or target tissue.

### Importance of accurate metadata

The term “metadata” is used to indicate all of the various data points that characterize the biological specimens collected. These data can be used to provide a critical context for the analysis of 16S rRNA sequences obtained from host-associated or environmental samples (National Research Council, 2007). Organizing and creating metadata to accurately reflect the data collected during an experiment are critical to bioinformatic and downstream analyses. There are multiple benefits to maintaining detailed metadata. Importantly, as much detail should be collected as possible to obtain adequate/accurate metadata, such as location of samples, dates, times, identification of sample source, single or pooled sample, treatments, or other observations made throughout the study. Once data are collected, the information should be appropriately stored in a consistent format, allowing for data to be easily understood by outside parties or collaborators. Some standards for metadata collection and formatting were described by Yilmaz et al. (2011); these standards should be followed especially if one intends to submit data to a publicly available database as is often required before publication (Yilmaz et al., 2011). Collecting accurate metadata improves downstream analyses in whichever bioinformatics program is used for diversity and taxonomic assignment and statistical analyses. Bioinformatic pipelines require consistency in titles and formats in metadata to appropriately input data, thus making accurate metadata important for analyses. Additionally, these sequence-processing pipelines often incorporate statistical analyses, which makes organizing metadata integral to make multiple comparisons among variables. While this can improve data observations, accurate metadata can also improve the reproducibility of collected data.

While not currently standardized across all fields, the push for standardized metadata to allow meta-analysis across 16S rRNA gene sequencing studies and reuse of sequenced data in other studies has been of increased interest. When metadata are standardized and accurate, large meta-analyses can more easily be conducted to detect underlying and large-scale trends not possible with one study alone (Dundore-Arias et al., 2020). In 16S rRNA gene sequencing studies, the best practice is to report some degree of metadata (many repositories will specify what metadata are required) in a repository with the published paper (typically along with raw data as talked about below). Therefore, organized metadata also assist researchers for publication purposes, reducing time spent on reorganizing the previously collected data.

A special consideration when collecting metadata in animal science research is if you are collaborating with industry partners (such as farms, pastures, processing facilities, or retail locations) that do not want to be identified. For example, some databases may ask for a geographical location of where samples were collected. It is essential that proper communication and nondisclosures are considered prior to the start of the study and what terminology can be used to describe the sampling location. In a manuscript, this could be nonspecific regional information (such as a commercial feedlot in the Midwestern United States) and in your publicly available metadata a geographic location that will not unblind a location such as the latitude and longitude of the university lab where it was processed.

Starting with organized and accurate metadata is critical for bioinformatic analyses of the data. Care should be taken in collecting data so that multiple sources may understand the metadata when conducting analyses. Overall, accurate metadata allow for ease in finding data, using the data in analyses, and then increasing the likelihood of data being used in future studies.

## Wet Lab Considerations

### Field handling and storage

Many factors can alter sample integrity during sample collection; thus, optimized sample preservation techniques for 16S rRNA gene sequencing are critical for accurate data (Carruthers et al., 2019). Immediately freezing (at least at −20 °C) a sample after field collection is the most ideal protocol, but in many cases, this is not possible for field studies. It has been documented that the length of time from collection to freezing can significantly alter 16S rRNA gene sequencing results. For example, one fecal study (Carruthers et al., 2019) investigated six different time periods between sample collection and freezing (1 to 36 h) and found that time to freeze was a significant factor affecting the results of microbiome composition. Additionally, a comparative storage study of feces (Moossavi et al., 2019) found that samples immediately frozen at −80 °C vs. those stored in 95% ethanol at room temperature prior to freezing displayed stability for certain bacterial populations, wheres other bacterial populations, such as *Actinobacteria*, were significantly altered. Nonetheless, in a comprehensive study of over 1,200 samples (Song et al., 2016), three different methods—95% ethanol, Flinders Technology Associates cards, and the OMNIgene Gut kit—all preserve samples sufficiently well at ambient temperatures. The effect of long-term storage at −80 °C has also been assessed and found that while changes will occur due to the length of storage time, the changes are similar to those seen in inter- and intra-subject variation and, therefore, remain acceptable for microbiome studies (Tap et al., 2019). Based on studies such as these, placing samples into sterile storage tubes and flash freezing in liquid nitrogen followed by transfer to a −80 °C freezer for immediate storage is likely to cause the least deviation from the “true” microbiome population. In some field work, liquid nitrogen or dry ice is not logistical possibilities, but care must be taken to get samples frozen as soon as possible to reduce 16S rRNA gene sequencing alterations as the bacterial community continues to live and change. In any case, care must be taken to treat samples uniformly across an experiment—that is, one day’s samples should not be left on the counter for more than 6 h with the next day’s samples being placed directly in the freezer.

Another critical factor that is often overlooked in 16S rRNA gene sequencing studies is the importance of a clean work area. Environmental contamination is a significant risk in agricultural settings and care must be taken to sterilize work areas. Surfaces areas in barns should be cleaned with ethanol or 10% bleach solution prior to sampling, and care should be taken to clean between samples to reduce cross-contamination. For example, soil samples left on a table surface can contaminate the next sample. While time consuming, the time taken to create a clean or sterile work area prior to sampling is important to reduce potential microbial contaminants. One of the elements of a clean sampling environment is the use of sterile sample swabs or collection bins. Not every brand or style is suitable for 16S rRNA gene sequencing work and care must be taken to purchase DNA-free, sterile swabs, bins, and tubes. Sterilization of workspace and timely uniform freezing practices will result in accurate, high-quality, 16S rRNA gene sequencing results.

### Lysis of cells

**Figure 3.**
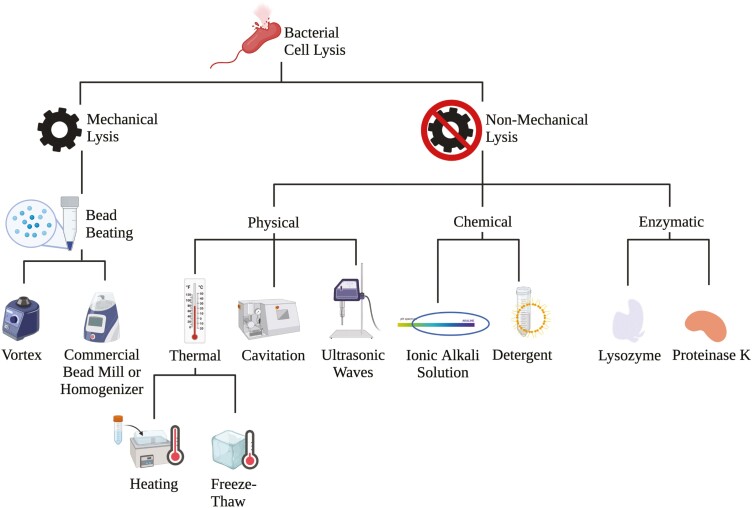
The common types of mechanical and nonmechanical lysis procedures utilized in animal science research applications. Created with Biorender.com.

To obtain representative quality genomic DNA to amplify the 16S rRNA gene and subsequently sequence the targeted region, the outer boundary, or cell wall and/or membrane, must be disrupted or lysed effectively. Cell lysis is the process in which the cell wall and/or membrane is disrupted or destroyed to release the inter-cellular material such as the DNA, RNA, protein, or organelles from the cell (Islam et al., 2017). There are currently multiple methods to disrupt the cell wall and/or membrane, including mechanical, physical, chemical, and enzymatic lysis (Figure 3). Mechanical lysis is common in animal science and is achieved through bead beating. Bead beating is often done using glass or zirconia beads with a commercial bead mill or vortex prior to employing a commercially available DNA extraction kit (Yu and Morrison, 2004; Qu et al., 2008; Danzeisen et al., 2011). Physical lysis can include freeze-thaw (Shao et al., 2012), heat, cavitation (Liu et al., 2020), and ultrasonic (Han et al., 2019) methods. Chemical lysis uses ionic alkali solutions or detergents to disrupt the bacterial cell membrane (Chapela et al., 2007; Martzy et al., 2019). Enzymatic lysis is accomplished through the application of enzymes such as proteinase k and lysozyme (Gill et al., 2016). These methods can be introduced individually, but most commercial DNA extraction kits such as the QIAamp PowerFecal Pro Kit (Qiagen, Valencia, CA, USA) utilize a combination of the aforementioned techniques to maximize lysis efficacy.

When considering which lysis technique, it is important to consider all sample types being compared in the study and what lysis steps will be appropriate to utilize throughout the study. There are two critical components to focus on when selecting a uniform lysis method: 1) will this method work on all of the cell types within the sample and 2) will this method work across all the sample types in the current study. Because some cells are more resistant to cell lysis, such as Gram-positive bacteria and bacterial endospores, the lysis technique selected could result in DNA that does not represent the true diversity or composition of the microbiome. As such, the mechanical bead beating step has been shown to improve DNA yield, increase observed bacterial diversity, and improve recovery of genomic DNA from Gram-positive and endospore-forming bacteria (Salonen et al., 2010; Maukonen et al., 2012; Guo and Zhang, 2013; Henderson et al., 2013; Walker et al., 2015). While not currently a concern for 16S rRNA gene sequencing studies on only one variable region, in the future, lysis methodologies capable of disrupting the cell wall and/or membrane without over fragmenting the genomic DNA will become more important as long-read sequencing technologies are more widely adopted.

To compare sample types properly, it is critical to consider all sample types and determine a standard lysis methodology, whether that be a single method or a combination of procedures. With inhibitors such as humic acid, bile salts, and polysaccharides being more prevalent among specific matrices (e.g., litter, feces, and feed), lysis procedures must account for these nuances. Furthermore, utilizing varying lysis methods between sample types could introduce bias into the analyses and result in differences in the subsequent microbiota populations that are not necessarily due to treatment or sample types (Henderson et al., 2013; Wesolowska-Andersen et al., 2014; Gerasimidis et al., 2016). Ma et al. (2020) investigated the effect of two chemical and two mechanical lysis methods on the 16S rRNA gene of the ruminal contents of goats and determined that the chemical lysis improved the DNA yield from fibrolytic bacteria such as Ruminococcaceae. However, they did note that chemical lysis methodology did not impact the number of unique bacteria detected. Ultimately, lysis methodology is an important parameter to consider when developing a study utilizing 16S rRNA gene sequencing. Depending on sampling type, there may be a need for optimization of lysis for DNA quality and quantity; this is especially true if you are working with a less common matrix in which case benchmarking DNA quality and output from different methods might be needed. Therefore, succinctly detailing the methodology utilized during the onset of a particular study is increasingly important as this can drive the differences seen between publications employing different lysis techniques.

### Low biomass samples

Certain agricultural samples, such as meat rinsates, water, environmental air, meconium, respiratory tract samples, reproductive tract samples, lavages, nasal swabs, or skin swabs, can be low in bacterial biomass, resulting in complications in DNA extraction and sequencing. Bacterial biomass is a critical determinant of sequencing output in amplicon datasets, and special care must be taken to optimize DNA extraction to prevent biased microbial profiles. Recent studies (Eisenhofer et al., 2019; Karstens et al., 2019) have investigated low biomass samples and found that lysing methods, DNA extraction, and PCR protocols could greatly influence results. An important first step in any 16S rRNA gene sequencing investigation is to read the literature on similar samples of interest to determine the best DNA extraction techniques for that specific sample type. A trial run on representative or pilot samples will ensure that DNA extraction methods are appropriate and yield reproducible results. Changes in protocols for DNA extraction are often needed, such as prolongment of lysing. A caveat of harsh mechanical or chemical lysing steps is the added concern of the quality of the DNA output as low-quality DNA can produce spurious PCR results, confounding data interpretation. While a larger quantity of DNA makes downstream processes easier, it should be noted that a suitable DNA extraction method can yield enough quality DNA from low bacterial biomass samples for 16S rRNA gene sequencing studies. One study has demonstrated that the bacteria present in single rumen protozoal cells could be taxonomically characterized using 16S rRNA gene sequencing (Park and Yu, 2018).

Other concerns with low biomass samples include introduction of outside microbes and low bacterial DNA yields. When sampling in a low biomass environment, sampling could introduce foreign microbes into the environment being sampled, such as exterior environmental microorganisms or resident vaginal microorganisms from a vaginal microbiome (Garcia-Grau et al., 2019). Future samplings may also be impacted due to colonization of the newly introduced microorganisms. Some microbiome sampling techniques have been developed specifically for this issue (see special considerations below). An additional issue may arise when you are sampling an environment with a low microbial load in relation to high host animal DNA presence—such as a meat rinsate. In this case, larger sampling amounts of pooling of several samples may be needed to obtain enough bacterial DNA (this is especially true for meat samples taken after interventions in processing facilities).

Once DNA extraction methods are optimized, PCR amplification can remain a complicating factor as low DNA levels can result in poor quality amplification or amplification of potential contaminants. Contamination becomes a greater concern in low biomass samples as low levels of pertinent microbes increase the potential for contaminating microbes to amplify significantly during the PCR step. The inclusion of controls, as discussed below, such as buffers or PCR-grade water, can help assess any potential contamination (Claassen-Weitz et al., 2020). Finally, if low biomass samples will not amplify efficiently, semi-nested PCR protocol instead of typical PCR may be used to increase PCR products. Increased PCR cycle numbers can also be used to increase amplicon yield but are associated with increased sequencing error rates and bias. Overall, low biomass samples can complicate 16S rRNA gene sequencing studies, but with careful experimental design and controls, these studies can yield reproducible results.

A final concern when sequencing DNA extracted from low biomass samples on an Illumina machine is that the low bacterial load and subsequent low GC content generated from these matrices generally impact the amount of Phi X (>10%) needed to produce a quality sequencing run and may also require that other samples with a more robust bacterial load be sequenced within the same library (Illumina Inc., 2021). 

### Library preparation and sequencing depth

An important decision one must make prior to the amplicon library preparation is which of the nine hypervariable regions of the rRNA gene will be amplified; common regions for analysis include V1-V3, V3-V4, or V4 (Figure 2). The V4 hypervariable region has been shown to allow adequate resolution and has been demonstrated to generate optimal community clustering with short-length reads (Caporaso et al., 2011) and has also gained popularity due to its use in the Earth Microbiome Project (Thompson et al., 2017). Regardless of variable region, sequencing instruments can produce single- or paired-end reads that allow for sequencing of the desired amplicons. Recent advancements in the sequencing chemistry used to produce short reads have been developed to allow for read lengths up to 300 base pairs (and around 500 bp if used paired-end reads), motivating researchers to sequence a combination of neighboring hypervariable regions. The advantage of doing this is that longer amplicons contain additional phylogenetic information that aids to resolve the taxonomic ambiguities present when assigning sequences to a taxon. The choice of hypervariable region(s) to sequence can be somewhat arbitrary but should ideally be driven by field- or research-specific reasons. For example, in arctic microbial communities, the V4-V5 hypervariable region is preferred because it provides superior taxonomic coverage and resolution of archaeal groups, which comprise a small, but an integral component of the arctic marine environment (Fadeev et al., 2021); in the female genital tract, the V3-V4 hypervariable region is preferred because it has the power to identify bacterial species associated with vaginal health and disease (Graspeuntner et al., 2018); in human skin, the V1-V3 hypervariable region is preferred because the microbial communities recovered approximate those reported from deep shotgun metagenomic assays (Meisel et al., 2016). In some cases, the choice of hypervariable region(s) may not be clear; in these cases, we recommend selecting a hypervariable region that is similar to those used in previous studies to which you may wish to compare your results.

Following amplicon primer pair selection to use for the library preparation, it is imperative that best practices in PCR preparation and handling should be conducted to minimize the occurrence of carry-over and cross-contaminations. While different steps of the library preparation process can have different locations in the laboratory, at minimum different pre- and post-amplification areas are needed, ideally in two different rooms or in PCR workstations/hoods. In addition to different workspaces, each area should be supplied with its own equipment (pipettes, tube racks, etc.) with items (including gloves and lab coat) not moving between the locations.

Another important decision to make in designing your 16S rRNA gene sequencing study is how many reads per sample you will aim for. Depending if the sequencing is occurring in-house or out-sourced, this could be as easy as specifying on a form the requested depth (such as 10,000 reads per sample) or may require a more complex calculation involving sample number, read length, and expected sequencing output. Some studies indicate as few as 2,000 reads allow characterization of a bacterial community (Caporaso et al., 2011), whereas others argue the number is closer to 10,000 to 15,000 (Bukin et al., 2019). The most appropriate method to find a minimum sequencing number target would be to construct a rarefaction curve (see the Diversity section for more on this) on a set of pilot samples. In the absence of any data on your matrix, 10,000 reads per sample would be a good starting point for a raw read number target.

### Inclusion of positive and negative controls

When preparing samples to sequence, the inclusion of positive and negative controls can identify any contamination in your wet-lab workflow and help in bioinformatic analysis. There are two types of negative control samples most commonly used in microbiome experiments: extraction controls and library controls. Extraction controls are employed during the extraction of nucleic acids and are typically composed of a lysis buffer, in the absence of any biological material. In the absence of any competing bacteria, these samples should have no cell to lyse; however, recent research into reagent and consumable contamination would indicate that this is not always true (Salter et al., 2014). The kits used to extract nucleic acids from biological samples also have their own microbiomes (termed the “kitome”) (Salter et al., 2014). In some cases, especially in low microbial biomass environments, these nucleic acids can outnumber those present in samples with real biological material. In extreme occasions, they can represent the entirety of the biological material being sequenced (de Goffau et al., 2019). Negative controls, when extracted alongside biological samples, allow the differentiation of meaningful biological differences from laboratory-acquired microbes. These can also be used to bioinformatically remove contamination sequences from the true samples, although this must be done carefully (Davis et al., 2018). Library controls (also known as no-template controls) are employed during the PCR amplification of the selected hypervariable region(s) and molecular-grade water, instead of DNA, is added. In the absence of any competing DNA, these samples should yield no or very little amplification. In many cases, this will likely hold true, as these samples are not exposed to the same conditions as extraction controls. Extraction and library controls should be included throughout the entire laboratory workflow and sequenced alongside samples containing the biological material being analyzed, even when they result in DNA quantification or amplification failures. Although the number and types of negative controls in a microbiome experiment can be vast, it will be up to the investigator to gauge which are most appropriate for ensuring the internal validity and confidence of study findings (Lipsitch et al., 2010).

There are many types of positive controls (i.e., mock communities) which are samples containing a known concentration and taxonomy of bacterial species. These controls, which can be made in-house in a lab or ordered commercially, can serve a wide range of applications, including as internal controls, measures of extraction efficiency, and measures of sequencing efficiency. *Internal controls* provide the technician with an assurance that the experiment was performed properly and that the original placement of samples was maintained throughout the entire laboratory workflow. *Extraction efficiency* can be measured using commercial mock communities containing a mixture of bacterial cells with both tough and easy-to-lyse cell walls (e.g., ZymoBIOMICS Microbial Community Standard, Cat No. D6300). These communities can provide an opportunity to measure the sensitivity of the extraction workflow, for example, the parameters used for mechanical cell disruption. *Sequencing efficiency* can be measured using commercial mock communities containing a mixture of bacterial cells present in various distributions, for example, a log distributed mock community, where the abundance of each bacterial species decreases by a power of 10 (e.g., ZymoBIOMICS Microbial Community Standard, Cat No. D6310). These communities can provide an opportunity to measure the sensitivity of the chosen sequencing depth to capture low abundant bacteria.

## Post Wet Lab Bioinformatics and Statistics

### Data and computational consideration

A computational analysis and data integrity plan should be in place prior to starting a 16S rRNA gene sequencing study. Computational considerations include the type of computer to be used, where data will be stored, how you will document your analysis, and how you will be doing the analysis (pipeline, web server, graphical user interface [**GUI**], etc.). The first consideration is if you will be performing the analysis on your local computer or remotely such as on a high-performance computing (**HPC**) cluster. Although it is possible to conduct a smaller study completely on a moderately equipped desktop or laptop, some of the more computational taxing steps (such as amplicon sequence variant [**ASV**] grouping and phylogenetic classification) might be limited to a larger machine. It is important to note that, after the initial steps of tree and feature table creation, it is likely that most downstream analysis can be conducted on a personal computer. Therefore, access to a moderately sized HCP for 3 to 5 d would be ideal (many universities now have HPC resources available to their students and staff for nominal fees or free and other resources exist such as Amazon Web Server).

Short-term data storage should also be considered. While long-term options for storage may be on a publicly available database (as described below), short-term storage is also important. Besides having enough room on a device, other considerations are the stability of the location (an external hard drive can be used but cloud storage is more reliable) and the accessibility. If you are using a shared server, make sure raw data are in a directory not easily editable. A good practice is to NEVER alter raw data files and have them stored in more than one location or on a mirrored drive. Documentation of your analysis is another facet that should be considered prior to the start of the experiment. Jupyter Notebook or GitHub provides a method to share analysis code and specifications which include important information such as versions of the tools used.

Finally, choosing the actual pipeline you will be using for your analysis is important. For 16S rRNA gene sequencing analysis, several different programs and tools will be used to generate your results, and all these tools together are called a pipeline. There are several well-developed pipelines for 16S rRNA gene sequencing analysis that allow the use of several tools wrapped into one either on the command line or through GUIs. When choosing what pipeline is best for you, price, user support, stability of the release, and your familiarity with the coding language (i.e., many of these require a basic familiarity with either Linux or Python languages) should all be considered. Popular 16S rRNA gene sequencing command line pipelines include QIIME2 (Bolyen et al., 2019), Mothur (Schloss et al., 2009), and RDP (Cole et al., 2014) (which are all free), whereas GUI options include Geneious and QIAGEN’s CLC Genomics Workbench (which both have a cost associated with them). As with any research tool, there are many technical and biological nuances to these analyses, and we strongly advise against using results without a cursory understanding of the methods used to come to your conclusions.

### Identifying contamination

Contamination, in the context of 16S rRNA gene amplicon sequencing, may be broadly defined as the detection of sequences not represented by microbial DNA in the originating sample. External contaminants may arise from laboratory workers (Adams et al., 2015), surfaces (Knights et al., 2011), and/or reagents (Salter et al., 2014). Despite the rigorous implementation of best practices, including aseptic technique, lab equipment sterilization, and reagent ultrapurification, the ubiquity of bacterial DNA in the environment can result in some, potentially nontrivial, level of external contaminant sequencing (Salter et al., 2014). Thus, in silico (performed on a computer) contaminant identification and removal approaches are important (Davis et al., 2018). Assuming no internal (i.e., cross-sample) contamination, the degree of primer specificity and template quantity are important considerations for in silico identification of potential contaminant sequences. Additionally, particularly for low biomass samples, the parallel amplification and sequencing of no-template controls (**NTC**s) are crucial for discriminating target sequences from nucleic acid extraction and PCR reagent contaminants.

Narrow taxonomic-range primers (e.g., genus- or species-specific probes) used to survey high-biomass samples provide the simplest scenario for in silico contamination detection and elimination. Following initial quality control steps (e.g., size selection, quality filtering, adaptor trimming, and chimera checking), single reads or contigs may be assigned taxonomically by alignment against a taxonomy database such as Silva, Greengenes, and RDP. Sequences with taxonomic assignments that fall out of the expected taxonomic range for the primer set may be discarded from further processing as nontarget. Accordingly, taxonomy-based contaminant identification steps are common in popular amplicon analysis pipelines, including QIIME2 (Bolyen et al., 2019), Mothur (Schloss et al., 2009), and DADA2 (Callahan et al., 2016). The user, based on probe specificity, may define a taxonomic range for sequence retention and further analysis. For example, even when using broad range “prokaryotic universal” 16S rRNA gene primers, any sequence classified as Eukaryotic may be culled as nontarget.

A more complex scenario for contaminant identification emerges when low-biomass samples are surveyed with broad taxonomy primers. Here, the combined effects of 1) low biomass, resulting in a lower number of target genes; 2) the presence of contaminant chromosomal DNA, ubiquitous in nucleic acid extraction and PCR regents; and 3) the potential for true overlap in the expected taxonomy of target and nontarget sequences are all confounding factors for in silico contaminant identification and removal (Salter et al., 2014). The common practice of removal of all overlapping sequences between NTCs and samples carries the risk of abundant true sequence removal due to the chance of legitimate taxonomic overlap between lineages commonly reported as kit contaminants and hardy survivors in low biomass environments (e.g., members of the spore-forming Firmicutes). This issue has been previously addressed by implementing custom analysis scripts that conservatively assess the probability of each taxon representing contaminants (Inagaki et al., 2015; Ramírez et al., 2019). Saliently, the decontam package, a frequency- and prevalence-based in silico contamination identification model for sequence features including 16S rRNA gene ASVs and operational taxonomic units (**OTU**s), accurately discriminated contaminants from the oral human microbiome and significantly reduced batch effects from a kit reagent- and sequencing center-driven contamination study (Davis et al., 2018). Designed on the dual assumptions that 1) contaminant DNA varies inversely with total DNA concentration in a sample and 2) contaminant sequences should be more common in NTCs relative to samples, decontam represents a significant, minimal or no cost, improvement for in silico contaminant identification. Due to decontam’s potential to significantly improve data quality and, consequently, biological interpretation based solely on a sequence feature frequency table, we strongly recommend its implementation as a standard practice in 16S rRNA gene sequencing of agricultural samples.

### Operational taxonomic units vs. amplicon sequence variants

For downstream analysis (both for taxonomy classification and diversity metrics), similar sequences are sorted into groups used as OTUs. Traditionally, 16S rRNA gene sequences have been clustered into OTUs differing by an arbitrary (often 3%) pairwise alignment dissimilarity threshold against all other sequences recovered from a single sample (de novo clustering) or against a set of reference sequences of an external sequence database (closed-reference clustering; Westcott and Schloss, 2015). Recently, however, the use of OTUs as the standard unit of 16S rRNA gene sequencing reporting has been challenged (Callahan et al., 2017). The clustering step of OTUs generation has important consequences for their biological interpretation and cross-study applications. There is no innate biological meaning to OTUs, that is, they are a “cloud” of similar sequences rather than a single species or genus. More specifically, OTUs are literal artifacts of 1) subjective dissimilarity threshold parameters in de novo clustering or 2) the database against which they are aligned in closed-reference clustering. Consequently, de novo OTUs cannot be compared between studies, and, in closed-reference clustering, biological information outside of the database is lost from the sequencing data.

Recently, the use of ASVs (also described in literature as Exact Sequence Variants) has been proposed as an alternative to OTUs (Callahan et al., 2016). ASV-based analyses distinguish sequencing errors from *bona fide* biological variation among 16S rRNA gene sequences using a model-based approach for correcting amplicon errors without constructing OTUs. Therefore, ASVs significantly reduce clustering artifacts and associated shortcomings of OTUs, thereby enabling valid comparison of ASVs independently generated from different samples (Callahan et al., 2016). Furthermore, ASV generation efficiently captures unique biological sequence variation, permitting exploration of signatures from all phylogenetic lineages in the dataset unrestricted by the limited variation present in even the most comprehensive closed-reference databases. Consequently, the replacement of OTUs by ASVs as the taxonomic unit for marker gene data analysis has been proposed (Callahan et al., 2017). One concern that has been brought up regarding ASV is the possibility of diversity inflation by the generation of multiple ASVs from a single bacterial genome (Schloss, 2021). Nonetheless, using ASVs allows cross-study tractability and database-independent biological veracity of AVSs, as discussed above, in addition to technical advantages in computational time and memory requirements. Specifically, as NGS technologies continue to improve and are applied to more large-scale agricultural and food safety monitoring, concomitant increases in the size of datasets are expected. By circumventing the need for sequence clustering (a computationally intensive step), ASVs can further enable routine large-scale microbial monitoring with relatively light and linearly scaled computational time and memory requirements.

### Accounting for uneven sampling depth

**Figure 4.**
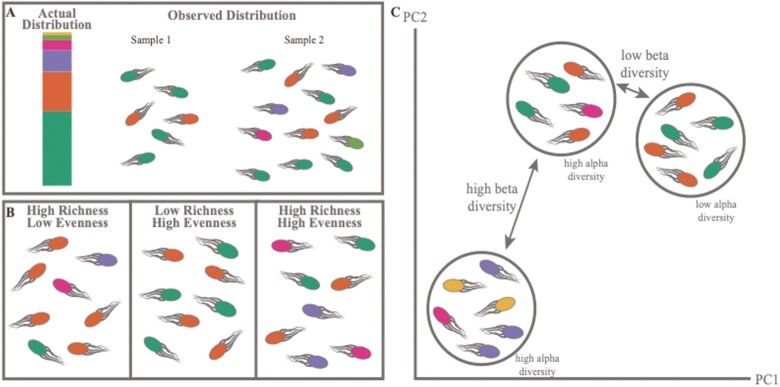
Illustration of considerations for diversity analysis. (A) Example of differences in sample composition based on sampling depth showing that different sampling depths between samples within an experiment can lead to false differences in diversity. This demonstrates the importance of using a normalization method before diversity analysis. (B) Illustration of communities that represent different features included in diversity metrics, specifically the relationship between richness and evenness in how diversity is calculated. (C) Demonstration of the differences in alpha and beta diversity. Alpha diversity represents the diversity within a sample and could be similar even in samples with different taxonomic compositions. Beta diversity describes the differences between samples and can only be calculated by comparing communities. This also demonstrates how samples can have similar alpha diversities but different beta dissimilarities.

Despite best practices during amplicon library preparation and sequencing, the numbers of raw reads generated from each sequencing run can vary widely among samples. This presents a problem for subsequent analysis, as uneven sampling can lead to artificially different diversity measurements between samples. Figure 4A demonstrates a hypothetical example of this problem, in which two samples that should appear similar because one sample has twice as many reads as another, which lets it capture more of the less abundant (“rare”) features. To combat this issue, normalization methods should be applied to the data before diversity analysis. Several normalization methods can be applied to amplicon sequence data, each with distinct advantages and disadvantages. Weiss et al. (2017) demonstrated that most methods result in correct clustering of samples in principal components analysis (see below for more information on this method), which indicates that all methods are valid tools and the researcher’s choice should depend on the specific circumstances of the dataset.

One commonly used method is to rarefy the data; that is, ASVs or OTUs within a sample are randomly subsampled without replacement to a preselected depth that is the same across all samples. The outcome of this is that all samples will have the same number of ASVs and any samples with fewer ASVs than the rarefaction level will be removed from the dataset. The level for rarefaction can be decided using a rarefaction curve, a method in which each sample is subsampled at multiple levels (e.g. 1,000 reads, 2,000 reads, 3,000 reads...), and the number of unique features or another metric of individual sample diversity of each sample at each level is measured and plotted. When the plot begins to level off after an initial climb up, the corresponding number of sequences indicates an appropriate sampling depth. The appropriate number to rarefy must then be balanced with the number of samples that may be dropped from the dataset which do not meet that minimum. An advantage of rarefaction is that it may be a more appropriate measure of very low-abundance (“rare”) ASVs. This can in turn increase the accuracy of the data, as low biomass samples often have contamination and quality concerns (Kennedy et al., 2014). There are also disadvantages to this method, the most obvious of which is the discarding of valuable data. Clearly, this is less than ideal as the researcher must pay for the samples and sequences, and in cases where the samples are very valuable or difficult to obtain the loss of data may be destructive to the overall experimental integrity. Additionally, the loss of statistical power by removing sequences from a sample could lead to a loss of differences between two samples (McMurdie and Holmes, 2014). The statistical consequences extend beyond this, as rarefying equalizes sample variance by adding artificial uncertainty (McMurdie and Holmes, 2014).

An alternative to subsampling data is to apply different types of transformation called normalizing, which is commonly used in other sequencing-based experiments such as RNA-seq and shotgun metagenomics. In this method, the ASV numbers are multiplied by a value or proportion, which can be determined through several specific methods. While early methods were less robust (Bullard et al., 2010; Dillies et al., 2013) (such as scaling by total count), more sophisticated normalization methods have emerged. Most are found within R packages built specifically for sequencing data. Some common methods include a median of ratios normalization from the DESeq2’s R package, Trimmed Mean of *M*-values (**TMM**) from EdgeR, and Cumulative Sum Scaling from the Metagenomeseq R package. In DEseq2, a scaling factor is computed as the median ratio of the count of an ASV over its geometric mean across all samples (Anders and Huber, 2010). In the TMM method, a sample is used as a reference and the weighted mean of log ratios of an ASV within a sample is compared with the reference (Robinson and Oshlack, 2010). Finally, in cumulative sum scaling, raw counts are divided by the cumulative sum of counts up to a percentile determined by the dataset (Paulson et al., 2013). These methods have been shown to be robust to differences between samples and maintain the differences in relative abundance (Dillies et al., 2013; McMurdie and Holmes, 2014). However, some transformations have been demonstrated to ignore or under-measure rare ASVs due to the impacts of the log transformation and can negatively impact the calculations of branch length in phylogenetic trees (Kennedy et al., 2014). Therefore, the choice between rarefying or using another transformation to normalize should depend on the value of individual samples and the importance of rare ASVs in a dataset; as such, a researcher should carefully consider the options for their particular dataset before selecting one.

### Alpha and beta diversity metrics

Diversity, either within one sample or compared between samples, is the measurement of how similar or dissimilar two measurements are. Calculating the diversity of bacteria within samples and comparing diversity between samples and treatment groups can be used to identify the changes that a treatment has caused to a microbial community or to understand why two microbial communities are inherently different. There are numerous methods by which to calculate and compare diversity metrics which can make approaching these analyses a formidable task for a new researcher. However, the methods can be readily categorized, which should enable a researcher to select the best options for their experiment (Figure 4B and C).

There are two overarching methods for evaluating diversity: alpha and beta diversity. Alpha diversity, or within-sample diversity, is used to evaluate the number of different species (usually represented by the number of ASVs) in each sample. The alpha diversity of a sample is fixed and does not change based on other samples to which it may be compared. This is different from beta diversity, or between-sample diversity, which is calculated as the dissimilarity between two samples and changes based on which samples are compared. To better understand the differences between alpha and beta diversity, it may help to consider the structure of the data. The alpha diversity can be added as a column to sample metadata and further analyzed the same as any other experimental condition. The beta diversity is structured as a distance or dissimilarity matrix, with the value of differences in diversity between each sample populating the cells.

Within both alpha and beta diversity, the methods for calculating the metric can be further subdivided depending on whether it is richness or evenness that are being evaluated (Figure 4B). Richness refers to the number of different species detected within a sample, regardless of how they are distributed. In Figure 4B, the first frame would have a richness of 4, as there are 4 distinct organisms present, even though the orange species is represented more frequently compared with the others. This distribution can be captured by measuring the evenness, or how balanced the species are within a sample. The second panel of the figure only has a richness of 2, but it is very even as the sample contains an equal of both species. The third panel represents a sample that is both rich and even, as the richness is again 4, but this time each species is equally represented. In alpha diversity calculations, the richness is captured by the observed OTUs/ASVs metric, evenness by Pielou’s evenness index, and both can be captured by Shannon’s diversity metric (Shannon, 1948; Pielou, 1966). In beta diversity, the richness is determined by a presence/absence metric, Jaccard distance, which calculates the number of shared species between two samples (Jaccard, 1901). The evenness in beta diversity is represented by the Bray–Curtis dissimilarity, which measures the fraction of overabundant counts between samples (Bray and Curtis, 1957). These methods are summarized in Table 2.

**Table 2.** Summary of the classifications and features of commonly used alpha and beta diversity metrics^1^

Metric	Alpha or Beta	Richness	Evenness	Phylogenetic
Observed features	Alpha	X		
Pielou’s Evenness	Alpha		X	
Shannon’s Index	Alpha	X	X	
Faith’s Phylogenetic Diversity	Alpha	X		X
Jaccard’s Distance	Beta	X		
Bray–Curtis Distance	Beta		X	
Unweighted UniFrac	Beta	X		X
Weighted Unifrac	Beta	X	X	X

1X indicates the metric includes this feature.

Another feature that can be incorporated into diversity metrics is phylogeny. Phylogenetic trees are representative of the evolutionary relationship between sequences in a sample and can be constructed de novo from only sequences in a dataset or compared with a reference tree via a fragment insertion method (Price et al., 2010; Eddy, 2011; Matsen et al., 2012; Janssen et al., 2018). The inclusion of phylogeny into a diversity metric allows a researcher to further investigate not only differences in diversity between samples but also how those differences are distributed evolutionarily, which may provide some insight into functional diversity. Phylogeny can be incorporated into alpha diversity using the Faith’s phylogenetic diversity index, which is a phylogenetic measurement of richness, and into beta diversity using the UniFrac dissimilarity, which can include just richness or richness and evenness depending on whether the unweighted or weighted method is used, respectively (Faith, 1992; Lozupone and Knight, 2005).

After diversity metrics are calculated, treatment groups can be statistically compared. Microbiome data are compositional and generally violate many assumptions in statistical analyses, especially that of normality, so nonparametric tests are used often. Moreover, given the number of pairwise comparisons that must be made to compare the samples, a multiple-testing correction should be applied to the *P*-value. There are several methods by which alpha diversity metrics can be compared, but most frequently the Kruskal–Wallis test is used to compare groups in discrete data and Spearman correlation is used to compare with continuous data (Spearman, 1904; Kruskal and Wallis, 1952). Alpha diversity comparisons are most frequently visualized using box plots (discrete) or scatter plots (continuous). In beta diversity, the statistics are slightly more complicated as they must be applied to the distance matrices. The permutational multivariate analysis of variance (PERMANOVA) test is frequently used when the data are univariate and categorical, and the Mantel test when it is univariate and continuous (Mantel, 1967; Anderson, 2017). Multivariate comparisons are also possible using the Adonis test (McArdle and Anderson, 2001). Beta diversity is also frequently evaluated using a clustering analysis, in which the dissimilarity between samples is reduced in dimensionality and plotted in either 2 or 3 dimensions using principal components analysis or principal coordinates analysis. In these plots, each point represents the entire microbiome of a sample, and the closer two samples are to each other the more similar their microbiome are. In Figure 4C, it is demonstrated how two samples with similar diversities, or a greater number of overlapping species, would reside close to each other in the space, while a sample with different species would reside separately. This figure also demonstrates how samples could have similar alpha diversities but a high dissimilarity when the beta diversity is calculated. This type of analysis is very useful for detecting trends in the data, but it is important to remember that it is not inherently representing any statistical significance. Overall, these tests are a very useful first step in analyzing the overall trends and differences in microbial communities, and, paired with other analyses, researchers can examine important aspects of the community and answer the driving research questions rather than just explore the community.

### Taxonomy

Another step in characterizing a microbiome is to determine its membership, which is traditionally done through a taxonomic analysis. Diversity metrics answer questions of how diverse a microbiome is and how microbiomes may be similar to each other, while taxonomy answers the question of who is there. Specifically, the taxonomy represents the identification and classification of each microorganism, represented by an ASV, present in the community. This is distinct from phylogeny, which represents evolutionary relatedness of the ASVs. In a typical taxonomic analysis, the representative sequences identified in the study are compared with a reference database that contains genome sequences and taxonomic information. This can be done simplistically by simply searching for the sequence in the database (McGinnis and Madden, 2004) or in a more complex manner, using machine learning algorithms to classify ASVs that may not be exactly represented by the database into a taxon (Bokulich et al., 2018). There are several well-curated databases available for researchers. The most popular include RDP (Cole et al., 2014), BLAST (McGinnis and Madden, 2004), SILVA (Quast et al., 2013), and GreenGenes (DeSantis et al., 2006). The choice of which is best for a given study should depend on how well represented the environment of interest is (e.g., the human gut is much more well described than the marine microbiome in GreenGenes) and how recently the database has been updated. When first deciding on which database to use, you can consider multiple databases and see which has a higher number of classified reads. The outcome of this analysis is a list that associates each ASV with a taxonomic label. These labels represent the highest-resolution level of taxonomy that can be achieved with confidence; in 16S gene sequencing, this often means that an ASV can only be classified to the family or genus level and not the species. The taxonomy in a study is generally represented with a stacked bar plot or a heatmap showing the relative abundance of an organism in each sample or group of samples.

### Differential abundance testing

Differential abundance testing is a statistical test used to identify specific taxonomic features that differ between two or more experimental groups. This is difficult to achieve in microbiome data as these data violate the statistical assumption of independence because all taxonomic features are expressed as relative abundance and a decrease in the abundance of one feature is accompanied by the increase in abundance of another to keep the sum as 100%. This means that, in a traditional statistical test, it would be impossible to determine whether the abundance of a feature was decreasing, or another was increasing. However, several methods can be employed to resolve this issue. If other analytical techniques, such as qPCR or flow cytometry, are used, the absolute abundance of one taxon can be determined (Vandeputte et al., 2017; Tkacz et al., 2018). However, in most cases, other analytical methods are not conducted in tandem with 16S rRNA gene sequencing; thus, this issue is addressed through downstream statistics. Several parametric statistical tests that were initially developed for RNA-sequencing analyses have been applied to microbiome studies, including DESeq2 and EdgeR, but they often fail to accurately represent microbiome communities as 16S rRNA gene sequencing data are sparse, meaning there are too many zeros in a dataset (Robinson et al., 2010; Love et al., 2014). More recent methods resolve the sparsity issue by including pseudocounts (i.e., adding 1 to every sample so there are no 0’s) and use specific normalization methods (e.g., log ratio normalization) to resolve the issues with feature distribution (Mandal et al., 2015). Some commonly used methods for differential abundance analysis include fitZIG, a zero-inflated Gaussian (ZIG) distribution mixture model (Paulson et al., 2013), ANCOM (Analysis of Composition of Microbiomes) which compares log ratios of abundance from each taxon to all the other taxa individually (Mandal et al., 2015), and a negative binomial model implemented in DeSeq2 (Love et al., 2014). In summary, a statistical comparison of the difference in relative abundance of individual taxa (e.g., genera or ASVs) between samples is statistically difficult, but ultimately bioinformatic tools specific to microbiome analysis have made this a useful analysis to include in microbiome studies.

### Data availability

Central to the reproducibility of an experiment is making the raw sequence data and other relevant metadata publicly available upon publication (Langille et al., 2018). In many cases, the bulk of the results and underlying conclusions are drawn directly from these data, so on-demand access should be expected (Langille et al., 2018). In fact, most journals now require raw data public availability, which we recommend as a best practice. Numerous online databases such as SRA (Leinonen et al., 2011) and EMBL (Hingamp et al., 1999) exist for this very purpose and even include standardized packages to describe and contextualize the submitted data. Taking advantage of these resources is paramount to ensure reproducibility of study findings, allowing quantitative comparisons across multiple studies and enabling the discovery of new findings. This is especially true for research fields that are just beginning to integrate these new tools into their research program. Equally important is the availability of the code used to derive meaning from these complex datasets. Numerous version control platforms (e.g., Github, GitLab, and BitBucket) exist for this very purpose and provide a convenient way for others to access, reproduce, and validate published study findings. However, the utility of these repositories is likely to be a function of the care and time used to produce them.

## Special Considerations

### Poultry

There are several poultry-specific caveats to consider before conducting 16S rRNA gene sequencing studies from poultry whether that be during production or processing. Specifically, the sample type used must be considered as these matrices can have a direct downstream effect on the 1) sequencing run and 2) the biological interpretations. In the past 10 yr, there have been numerous efforts to elucidate the microbiome of poultry samples representative of those typically collected by the U.S. Department of Agriculture’s Food Safety Inspection Service (**USDA-FSIS**) and Hazard Analysis and Critical Control Point (**HACCP**) personnel for process control and biomapping (Rothrock et al., 2016; Kim et al., 2017; Handley et al., 2018). As the sampling methods utilized by USDA-FSIS and HACCP personnel are not aimed at 16S rRNA gene sequencing but rather traditional confirmation techniques, the direct extraction of the genomic DNA from these matrices can be difficult and may require additional lysing steps such as mechanical bead beating (Feye and Ricke, 2019). Low biomass samples, such as whole bird carcass rinses (Kim et al., 2017; Handley et al., 2018), scalder and tank water (Rothrock et al., 2016), skin and feather rinses (Rothrock et al., 2019), can not only be hard to isolate sufficient quantities of DNA from, but, due to the insufficient bacterial load, can also result in additional filtration and lysing steps to maximize DNA recovery.

Additionally, the area within the poultry house or environment must be considered as there are distinct differences in the litter and feces due to the house structure (feeders, drinkers, evaporative cool pads, ventilation fans). Locatelli et al. (2017) demonstrated that location within poultry houses does matter in terms of the microbiota collected from the feces and litter at the fans, cooling pads, waterer/feed lines, and the bulk litter areas, as well as manually and in silico pooling of these samples did not yield equivalent fecal microbiota compositions. As such, research must consider the differences between matrices to appropriately identify which matrix is most appropriate for their hypothesis.

### Bovine reproduction tract microbiomes (uterine/vaginal)

A recent implementation of 16S rRNA gene sequencing in bovine research has been focused on reproductive efficiency due to the discovery of the reproductive tract microbiome. Previously, the presence of microorganisms in the reproductive tract was believed to only indicate disease and reproductive failure in humans and animals. However, 16S rRNA gene sequencing has provided the opportunity to detect a microbiome within the uterus and vagina, which is largely dominated by the presence of unculturable microorganisms. The uterine and vaginal microbiome have a significantly lower microbial biomass than other body locations across multiple species, such as the GIT (Huttenhower et al., 2012; Swartz et al., 2014). Nonetheless, by the use of 16S rRNA gene sequencing, the Human Microbiome Project reported that the urogenital microbiome contains only 9% of the body’s total microorganisms (NIH HMP Working Group et al., 2009). As a low biomass environment, best practices for low biomass sampling like discussed previously must be followed. Common methods for microbial sampling of the reproductive tract include the use of protected sterile swabs or flushing saline through a sterile catheter. Insertion of the catheter or swab through a sheath or speculum-like guard will reduce the potential of introducing exterior or vaginal microorganisms into the uterus, or accidental collection of these microorganisms in the sample. The inclusion extraction controls such as collected open-air tubes can help determine the potential contaminant microorganisms that may be present in the sample (Eisenhofer et al., 2019; Karstens et al., 2019). Best practices in sampling to reduce contaminants and the inclusion of controls will help ensure reproducible and reliable study results for the reproductive microbiome.

## Conclusion

When best practices are followed, 16S rRNA gene sequencing can provide ecological insights not afforded by traditional microbiology methods alone. There are microbiome-specific concerns in all phases of a study including planning, wet lab, bioinformatics, and statistical analysis, but with proper planning, these concerns can be addressed, and issues mitigated. When questions do arise regarding any specific portion of an experiment, the first question should be how it relates to your specific hypothesis as no answer is one size fits all; reviews of the literature closely related to your study or other scientists doing similar work are appropriate sources of solutions. When beginning to study a new ecological niche with little-to-no background information, a small pilot study of a few samples will answer many questions on sequencing depth and sampling location. Nonetheless, following the outlined best practices included here will set a solid foundation to build your microbiome research on.

## Abbreviations

ASV: amplicon sequencing variant
GIT: gastrointestinal tract
GUI: graphical user interface
HACCP: Hazard Analysis and Critical Control Point
HPC: high-performance computing cluster
NGS: next-generation sequencing
NTC: no template control;
OTU: operational taxonomic unit
TMM: trimmed mean of *M*-values
USDA-FSIS: U.S. Department of Agriculture’s Food Safety Inspection Service
